# A DNA repair defect in a radiation-sensitive clone of a human bladder carcinoma cell line.

**DOI:** 10.1038/bjc.1992.171

**Published:** 1992-06

**Authors:** S. N. Powell, S. J. Whitaker, S. M. Edwards, T. J. McMillan

**Affiliations:** Radiotherapy Research Unit, Institute of Cancer Research, Sutton, Surrey, UK.

## Abstract

DNA repair was measured in an ionising radiation-sensitive mutant of a human bladder carcinoma cell line. No difference in the rate or extent of double-strand break rejoining was found using the techniques of neutral filter elution and pulsed-field gel electrophoresis. In contrast, significant differences in repair fidelity, measured by plasmid reconstitution, were found. The parent line had a repair fidelity of 84.7% compared with 58.9% for S40b (P = 0.0003). It is suggested that repair fidelity can be an important determinant of radiosensitivity in human tumour cells.


Br. J. Cancer (1992), 65, 798–802 © Macmillan Press Ltd., 1992

### A DNA repair defect in a radiation-sensitive clone of a human bladder carcinoma cell line

S.N. Powell, S.J. Whitaker, S.M. Edwards & T.J. McMillan

*Radiotherapy Research Unit, Institute of Cancer Research, Cotswold Road, Sutton, Surrey SM2 5NG, UK.*

**Summary** DNA repair was measured in an ionising radiation-sensitive mutant of a human bladder carcinoma cell line. No difference in the rate or extent of double-strand break rejoining was found using the techniques of neutral filter elution and pulsed-field gel electrophoresis. In contrast, significant differences in repair fidelity, measured by plasmid reconstitution, were found. The parent line had a repair fidelity of 84.7% compared with 58.9% for S40b ($P = 0.0003$). It is suggested that repair fidelity can be an important determinant of radiosensitivity in human tumour cells.

The isolation of mutants with either increased or decreased sensitivity to cytotoxic agents has proved to be invaluable in the elucidation of mechanisms of action of such agents. The development of ionising radiation sensitive mutants had been largely limited to variants of Chinese hamster cells and the L5178Y murine lymphoma cell line (Sato & Hieda, 1979; Jeggo & Kemp, 1983; Giaccia *et al.*, 1985; Jones *et al.*, 1988). Mechanistic studies of the nature of radiation sensitivity in human cells has been mainly limited to the study of ataxia-telangiectasia (A-T) cells. To increase the available human lines we have previously isolated an ionising radiation-sensitive clone (S40b) of a human bladder carcinoma cell line MGH-U1 (McMillan & Holmes, 1991).

DNA repair deficiencies have been described for some of the radiosensitive rodent cell lines. The *xrs*, XR-1 and L5178Y-S mutants have been reported to show defective double-strand break (dsb) rejoining (Kemp *et al.*, 1984; Giaccia *et al.*, 1985; Evans *et al.*, 1987; Wlodek & Hittelman, 1987) measured by neutral filter elution. The *irs* mutants have no measurable impairment of dsb rejoining, but *irs1* was found to have low repair fidelity compared with its parent line, V79, using plasmid reconstitution (Debenham *et al.*, 1988*b*). *Irs2* has been reported to exhibit radioresistant DNA synthesis (Jones *et al.*, 1990). Low repair fidelity and radioresistant DNA synthesis are both reported features of A-T (Cox *et al.*, 1986; Debenham *et al.*, 1988*a*; Painter, 1981). A-T cells have no defect in dsb rejoining (Lehmann, 1982) with one reported exception (Coquerelle *et al.*, 1987).

In a previous report (McMillan & Holmes, 1991), the initial DNA damage, measured by neutral filter elution, was found to be the same in clone S40b and the parent line, MGH-U1. To investigate the possibility of a repair defect in S40b we have now looked at DNA repair under two broad headings: (1) the rate and extent of dsb rejoining (2) repair fidelity. Dsb rejoining was assessed by neutral filter elution (NFE) and pulsed-field gel electrophoresis (PFGE). Repair fidelity was measured by plasmid reconstitution.

### Materials and methods

### *Cell lines*

The parent line, MGH-U1, and a radiosensitivie clone, S40b, were grown in Ham's F12 medium supplemented with 10% foetal bovine serum, streptomycin (100 mg $l^{-1}$) and penicillin ($10^5$ units $l^{-1}$) as an attached monolayer. All cultures were incubated at 37°C in 3% $O_2$, 5% $CO_2$ and 92% $N_2$. The difference in cell survival following ionising radiation is shown in Figure 1 (from McMillan & Holmes, 1991). The radiosensitivity of S40b has been observed to be stable over 18 passages.

### *Neutral filter elution (NFE)*

The technique of Bradley and Kohn (1979) with the modifications detailed in McMillan *et al.* (1990) was used. Briefly, test cells were labelled with 0.075 $\mu$Ci ml$^{-1}$ $C^{14}$ methyl-thymidine and internal standard MRC5 CV1 cells were labelled with 0.086 $\mu$Ci ml$^{-1}$ $H^3$ methyl-thymidine, both for 30 h. The medium was replaced 15 h before assay by fresh non-radioactive medium. The MRC5 CV1 cells were treated with 100 Gy. For repair studies, cells were irradiated to 50 Gy in monolayer culture, and maintained at 37°C for 1, 2 and 4 h after irradiation. At the end of the repair time, medium was replaced with ice-cold phosphate buffered saline, and the cells were harvested by mechanical disaggregation using a rubber policeman. Aliquots containing $1-2 \times 10^5$ test cells mixed with $10^5$ MRC5 CV1 cells were gently sucked onto pre-wetted, ice-cold polycarbonate filters (2 $\mu$m pore size, Nucleopore) contained in Swinnex filter units (Millipore). Cells were lysed on the filter for 15 min in elution buffer, containing 50 mM Tris, 50 mM glycine, 25 mM disodium EDTA, and 20 g $l^{-1}$ of sodium lauryl sulphate at pH 9.6. DNA fragments from the lysed cells were eluted at a flow rate of 2 ml $h^{-1}$ for 16 h, with individual samples collected over 100 min. At the end of elution, DNA fragments remaining on the filter were released by treatment with 0.8 ml of 1 M hydrochloric acid at 60°C for 1 h. 0.4 M NaOH was added and shaken vigorously to restore neutral pH and 1 h later scintillant (Picofluor-40, Canberra Packard) was added to all samples. Scintillation counting was performed in a Canberra Packard tricarb 2000 CA scintillation counter with correction for luminescence and spill-over in the tritium channel. The fraction of test cell DNA retained when 40% of MRC5 CV1 DNA was retained was used for analysis. The relative elution of the test DNA was derived by subtracting the proportion of DNA eluted for unirradiated cells from that eluted from test cells. The repair experiments are presented as the proportion of the time zero relative elution value which is recovered after various repair times, within one experiment. Four independent experiments were performed to obtain the percent of dsb repaired at each time point.

### *Pulsed field gel electrophoresis (PFGE)*

PFGE was used as an alternative means of measuring DNA fragmentation using a CHEF-DRII system (Bio-Rad) as described previously (Whitaker & McMillan, 1992). Expontentially growing cells were labelled with 0.05 $\mu$Ci ml$^{-1}$ $C^{14}$

Correspondence: S. Powell, Department of Radiation Oncology, Massachusetts General Hospital, Boston, Massachusetts 02114, USA.
Received 27 August 1991; and in revised form 9 January 1992.

**Figure 1** The high dose-rate (1–2 Gy min) single dose, $^{60}$Co radiation cell-survival curves for MGH-U1 and the radiosensitive clone S40b. The solid lines represent a non-linear regression using the linear-quadratic model.

thymidine for 48–72 h and fresh non-radioactive medium added for 15–18 h prior to irradiation. Cell irradiation to 20 Gy was carried out in monolayer at 37°C. After allowing the specified time for repair following irradiation, culture medium was replaced with ice-cold phosphate buffered saline and cells were harvested at 4°C using 0.02% versene. The cells were washed and pelletted at 4°C then resuspended in 0.8% ultra-low melting point agarose at 18°C. The cells ($2 \times 10^6$ ml$^{-1}$) in agarose suspension were placed in ice-cold plastic moulds, and incubated at 4°C until the agarose had set. The 'cell plugs' were placed in ice-cold lysis solution containing 0.5 mg ml$^{-1}$ proteinase-K (Boehringer-Mannheim) in 2% lauroyl-sarkosine (Sarkosyl, Sigma) and 0.5 M EDTA at pH 7.6. Plugs were held on ice for 1 h to allow permeation of EDTA, designed to prevent repair which might occur on warming. Plugs were then incubated at 37°C for 18 h. The rapid action of detergent will also prevent further repair at this temperature. The plugs were used within a few days, because long-term storage causes a significant increase in background damage due to the incorporated $C^{14}$. This technique is similar in principle to that used by Blöcher *et al.* (1989) and Stamato and Denko (1990).

Cell plugs of approximately 25 µl (containing $5 \times 10^4$ cells) were loaded into the wells of a 0.8% agarose gel. The CHEF-DRII unit delivers two homogeneous electric fields at +60° and −60° to the direction of net movement. Parameters for electrophoresis were 3.6 V.cm$^{-1}$, 60 min field switch time with a total running time of 96 h. Electrophoresis buffer (0.5 × TBE) was maintained at 16°C.

Damage was measured in an analogous manner to NFE. The proportion of DNA which moved out of the well and into the gel relative to the total DNA in the lane was recorded as the measure of damage. This proportion for unirradiated cells was 2–5% and was subtracted from the value for irradiated samples. Repair was also recorded as for NFE: the per cent of the initial level of damage. Each time point at 1, 2 and 4 h reflects data from three or four independent experiments. Data were also obtained for MGH-U1 at 1 h and 3 h from two experiments.

*Plasmid reconstitution*

A plasmid, pPMH16 (kindly donated by Dr J. Thacker, MRC Radiobiology Unit, Chilton, Oxon) with two selectable bacterial genes was used for transfection in the repair assay (Debenham *et al.*, 1988*a*). The first gene (*neo*) confers resistance to the antibiotic G418, a derivative of neomycin which crosses the mammalian cell membrane. The second gene (*gpt*) confers resistance to the medium XHATM (xanthine, hypoxanthine, aminopterin, thymidine and mycophenolic acid) by utilising xanthine to make guanine whose production is otherwise inhibited (Mulligan & Berg, 1981). No function of these genes occurs naturally in mammalian cells: when present they must be derived from transfected DNA. Both genes have mammalian cell promoters immediately upstream and mammalian signalling sequences downstream to ensure expression following transfection (Debenham *et al.*, 1988*a*).

The principle of the assay is to use the *neo* gene as a marker of transfection, and to damage the *gpt* gene using a restriction endonuclease, which causes a double-strand break (dsb) in the plasmid. The ability of the transfected cells to reconstitute the damaged *gpt* gene and to restore its function is tested.

*Plasmid digestion by restriction enzyme*

Restriction enzyme, *Kpn*I, cleaves the plasmid once within the *gpt* coding region to produce a linear plasmid with 'cohesive' ends. Plasmid was digested prior to transfection with 2 units of enzyme per µg plasmid for a minimum of 3 h. The linearity of the plasmid was confirmed using gel electrophoresis (0.8% agarose in Tris-Borate-EDTA).

*Plasmid transfection*

The transfection procedure was as follows: 24 h prior to transfection, $10^6$ cells were seeded into an 80 cm$^2$ flask; 2 h prior to transfection the medium was replaced with 5 ml of fresh medium; for transfection, 40 µg of plasmid DNA (cleaved or circular) in 1 ml of Hanks buffered saline (HBS) with 125 mM $CaCl_2$ was added for an exposure of 6 h. The cells were then washed with medium without serum, exposed to 15% glycerol in 1.5 ml of HBS for 3 min, and washed again before replacing complete medium. A further 48–60 h was allowed (expression time) before harvesting the cells using trypsin (0.05%)/versene (0.02%) and seeding the cells, without feeder cells, at $5 \times 10^5$ or $10^6$ per 80 cm$^2$ flask (2–3 per experiment) in medium containing 0.5 mg ml$^{-1}$ G418. Cell viability following the transfection procedure was 65% for MGH-U1 and 50% for S40b. This is 80–90% of normal plating efficiency. After 10–14 days growth in G418 (medium changed every 7 days) visible viable colonies could be marked. Selection for *gpt* gene function was then applied with medium (XHATM) containing xanthine (10 µg ml$^{-1}$) hypoxanthine (13.6 µg ml$^{-1}$) aminopterin (0.176 µg ml$^{-1}$) thymidine (3.87 µg ml$^{-1}$) and mycophenolic acid (10 µg ml$^{-1}$). The proportion (P) of marked colonies which remained viable in XHATM after 7 days was recorded.

*Repair fidelity*

Repair fidelity was defined as: P (cleaved plasmid) ÷ P (circular plasmid). Repair fidelity represents the efficiency of accurate rejoining of the double-strand break (dbs) by the tested cell line. The denominator takes into account the probablity that the integration of previously undamaged plasmid may damage for *gpt* gene. The assay thus focuses on the correction of the cleaved plasmid. Repair fidelity values for the sensitive clone and the parent line were compared using an unpaired *t*-test on logarithmically transformed data.

*Irradiations*

Cells were irradiated using a 33 TBq (900 Ci) $^{60}$Co source at a dose rate of 1–2 Gy min$^{-1}$.

### Results

*Double-strand break rejoining*

The data obtained for dsb rejoining using neutral filter elution are shown in Figure 2. The initial rate of rejoining was equal for the two cell lines, with both achieving 60% rejoining after 1 h. However, between 1–4 h little rejoining was discernible within the error of the data. At 4 h rejoining was no more than 70% complete. Thus, within the number of time-points investigated, no difference in the rate or extent of double-strand break removal could be detected.

The data obtained using pulsed-field gel electrophoresis is shown in Figure 3. The same time-points of repair were investigated. In essence, a similar result was observed. No significant difference in the rate or completeness of rejoining was found between the sensitive clone and the parent line. Compared with the data obtained using neutral filter elution, marginally greater rejoining was observed. At 1 h the damage was 70% rejoined, and at 4 h damage was 80% rejoined.

### *Repair fidelity*

The mean transfection frequency for MGH-U1 was 1.58 × $10^{-4}$ in G418 selection and 2.17 × $10^{-4}$ directly in XHATM selection using circular plasmid. For *Kpn*I-cleaved plasmid the values were 1.16 and 0.15 × $10^{-4}$ respectively. For S40b, the transfection frequency with circular plasmid was 8.8 × $10^{-4}$ in G418 and 13.6 × $10^{-4}$ directly in XHATM. The corresponding figures for *Kpn*I-linearised plasmid were 14.2 and 0.57 × $10^{-4}$. Thus, transfection frequency was higher in the sensitive clone and this difference was maintained in experiments performed at the same time with the same plasmid DNA solutions.

**Figure 2** Double-strand break rejoining measured by neutral filter elution for MGH-U1 and the radiosensitive clone S40b. Damage repair is plotted as the % decrease in the initial level of damage with time. Error bars are standard error of the mean.

**Figure 3** Double-strand break rejoining measured by pulsed-field gel electrophoresis for MGH-U1 and the radiosensitive clone S40b. Damage repair is plotted as the % decrease in the initial level of damage with time. Error bars are standard error of the mean.

The repair fidelity values of MGH-U1 and clone S40b are shown in Table I. Both cell lines showed very similar proportions of XHATM-resistant colonies among the G418-resistant colonies when undamaged plasmid was transfected into the cells (column A). These values are reduced to a greater extent in S40b when the *KpnI* cut plasmid was used, resulting in a significant reduction in the overall repair fidelity. The geometric mean repair fidelity for *KpnI*-cleaved plasmid was 84.7 for MGH-U1 and 58.9 for S40b ($P = 0.003$).

### Discussion

It has been reported that a deficiency in double-strand break removal is responsible for the radiosensitivity of the *xrs* and XR-1 mutants of Chinese hamster ovary cells and the L5178Y-S mouse lymphoma mutant (see review: Jeggo, 1990). However, this is not a universal feature of radiosensitive cells and indeed in the present study we have been unable to detect such a deficiency in S40b.

The repair kinetics up to 1 h were not evaluated in this study. Measures of repair at 30 min have been obtained for MGH-U1, but they were not obtianed in parallel with S40b. In these unpublished studies, repair at 30 min was 84, 92 and 100% of the value at 1 h from three independent experiments. The expected error of these data points is large because there is a rapid change in the amount of damage with time. The power to discriminate small changes in the initial repair kinetics with these techniques is limited. Only when the differences are large (e.g. *xrs* compared with CHO-K1; Kemp *et al.*, 1984) can the initial repair kinetics be clearly distinguished, and this is always accompanied by a clear difference in the level of residual damage. Schwartz *et al.* (1988) have claimed that damage repair at 1 h (after 100 Gy) allows discrimination of radioresistant from radiosensitive tumour cell lines, but the magnitude of the difference was small. This study only assessed damage at 1 h and 2 h, and measured the amount of damage remaining rather than the percentage repair. It was equally clear in these data that the initial level of dsb was higher in the radiosensitive lines. Thus far, differences in initial repair kinetics, without differences in the residual level of damage have not been clearly demonstrated.

Although we did not examine repair times of less than 1 h it appears that the kinetics of double-strand break removal observed using either NFE or PFGE are similar, since repair was essentially complete within 1 h. This similarity between the two techniques suggests that the values reflect a biological parameter rather than a measure which is dependent upon the technique. The repair kinetics found in this paper are in the range previously reported for NFE (10–40 min

**Table I** The repair fidelity of MGH-U1 and its radiosensitive clone S40b

| *Cell line* | *Expt No.* | *Undamaged plasmid (A)* | *KpnI cut plasmid (B)* | *KpnI repair fidelity (%) (C)* |
|---|---|---|---|---|
| MGH-U1 | 1 | 0.92 (61/66) | 0.76 (46/60) | 82.3 |
| | 2 | 0.9 (9/10) | 0.71 (15/21) | 79.3 |
| | 3 | 0.9 (9/10) | 0.75 (3/4) | 83/3 |
| | 4 | 0.95 (39/41) | 0.89 (71/80) | 93/3 |
| | 5 | 0.94 (76/81) | 0.81 (72/89) | 86.2 |
| S40b | 1 | 0.95 (60/63) | 0.57 (80/141) | 59.6 |
| | 2 | 0.91 (52/57) | 0.58 (19/33) | 63.1 |
| | 3 | 0.91 (81/89) | 0.5 (66/133) | 54.5 |

Columns A and B show the proportion of G418 resistant colonies which are also XHATM resistant for circular and *KpnI*-cleaved plasmid respectively. Column C is the calculated repair fidelity. The *KpnI* repair fidelity for MGH-U1 and S40b are significantly different ($P = 0.0003$).

half-time, Bradley & Kohn, 1979). The similarity of the results obtained using filter elution and PFGE are in contrast with the measurements of repair half-time using velocity sedimentation (e.g. Bryant & Blöcher, 1980) where values of 1.5–4 h were found. The reason for this discrepancy remains unclear.

Both measures of damage repair show significant residual damage (20–30%) at 4 h. The time course of rejoining has not been followed to longer times to ascertain whether complete rejoining occurs. The data from NFE are derived after 50 Gy radiation dose compared with 20 Gy for PFGE. The higher level of residual damage found with the filter elution studies suggest this may be a dose dependent phenomenon. However, the level of residual damage is the subject of our continuing study, and at this stage we cannot exclude that the level of residual damage may be in part dependent upon the technique of measurement. Differences in the final amount of damage may not be detected because of the inadequate sensitivity of the strand-breakage studies, although using a combination of measurement methods and the advent of PFGE we would hope to increase the sensitivity.

Our results suggest that it may be misrepair, rather than lack of dsb rejoining, which is the basis of radiosensitivity in S40b. The only significant difference in DNA repair between the radiation sensitive clone and its parent line was in repair fidelity. We have shown that the repair fidelity of the *KpnI*-cut plasmid was 84.7% for MGH-U1 and 58.9% for S40b. Reduced repair fidelity has been previously seen in other radiosensitive variants including A-T fibroblasts (Cox *et al.*, 1986), *irs1* (Debenham *et al.*, 1988*b*) and a sensitive clone from a human glioma cell line (Powell & McMillan, 1991). It is suggested that repair fidelity may be an important determinant of radiosensitivity. It is also possible that decreased repair fidelity is also reflected in an increased mutation rate and this is currently under investigation.

An important consideration in these experiments is that a restriction endonuclease-induced break may be chemically similar to only a small proportion of radiation-induced dsb. However, radiation and restriction endonucleases (introduced directly into cells) produce similar biological effects (Obe *et al.*, 1986; Bryant, 1988), and some radiation sensitive mutants have been shown to be sensitive to restriction endonucleases (Barnes & Rhine, 1985; Bryant *et al.*, 1987). It seems, therefore, that the dsb induced by radiation and restriction endonucleases may be processed in similar ways.

The burden of damage the cells require to repair within the two types of assay (repair fidelity *vs* strand-break rejoining) is not possible to quantify. Many millions of plasmids are introduced into the cell culture medium, but between the initial step and measurement of the number of integrated plasmids, precise quantification is not possible. Radiolabelling of transfected plasmids has suggested cellular uptake by significant numbers of plasmids (thousands) but distinguishing an intracellular location from attachment to the cell membrane is difficult (J. Thacker, personal communication). Using different amounts of transfected plasmid (40 μg *vs* 1 μg) which led to different numbers of integrated plamids (5–10 *vs* 2–3 respectively) there was no difference in the measured repair fidelity. This implies that repair fidelity is a measure of accuracy which is independent of the number of repair events. These unpublished observations are available only for MGH-U1.

The physical nature of the misrepair has not been examined in the present study. However, our previous work using a sensitive clone of a glioma cell line, showed that low repair fidelity was associated with misrepair of the *gpt* gene (Powell & McMillan, 1991). Using Southern analysis, the number of intact *gpt* gene copies relative to the number of plasmids integrated was found to be lower in the sensitive clone. In other cell lines we have shown that the misrepair may result in small changes (undetectable using Southern analysis) or large deletions where significant sections of the *gpt* gene and the surrounding DNA are lost. Whether it is increase endonuclease activity, a lack of protection of exposed termini or abnormal recombination which leads to these changes, is not clear. Due to the size of some of the deletions and the demonstration by Folger *et al.* (1982) that integration of linear plasmid is probably by a recombination step rather than prior religation, we favour a recombination defect as the likely explanation of our results.

We are grateful to Professor G.G. Steel and Mr J.H. Peacock for advice during this work and in the preparation of this mansucript. Miss R. Couch and Mrs S. Stockbridge provided invaluable secretarial assistance. This study was supported by the Cancer Research Campaign and the Bob Champion Cancer Trust.

### References

BARNES, G. & RINE, J. (1985). Regulated expression of endonuclease EcoR1 in *Saccharomyces cerevisiae*: nuclear entry and biological consequences. *Proc. Natl Acad. Sci. USA*, **82**, 1354–1358.

BLOCHER, D., EINSPENNER, M. & ZAJACKOWSKI, J. (1989). CHEF electrophoresis, a sensitive technique for the determination of DNA double-strand breaks. *Int. J. Radiat. Biol.*, **56**, 437–448.

BRADLEY, M.O. & KOHN, K.W. (1979). X-ray induced DNA double strand break production and repair in mammalian cells as measured by neutral filter elution. *Nucleic Acids Res.*, **7**, 793–804.

BRYANT, P.E., BIRCH, D.A. & JEGGO, P.A. (1987). High chromosomal sensitivity of Chinese hamster xrs5 cells to restriction endonuclease-induced DNA double strand breaks. *Int. J. Radiat. Biol.*, **52**, 537–554.

BRYANT, P.E. (1988). Use of restriction endonucleases to study relationships between DNA double-strand breaks, chromosomal aberrations and other end-points in mammalian cells. *Int. J. Radiat. Biol.*, **54(6)**, 869–890.

BRYANT, P.E. & BLOCHER, D. (1980). Measurement of the kinetics of DNA double-strand break repair in Ehrlich ascites tumour cells using the unwinding method. *Int. J. Radiat. Biol.*, **38**, 335–347.

COQUERELLE, T.M., WEIBEZAHN, K.F. & LUCKE-HUHLE, C. (1987). Rejoining of double strand breaks in normal human and ataxia-telangiectasia fibroblasts after exposure to 60-cobalt gamma-rays, 241-Am alpha particles or bleomycin. *Int. J. Radiat. Biol.*, **51**, 209–218.

COX, R., DEBENHAM, P.G., MASSON, W.K. & WEBB, M.B.T. (1986). Ataxia-telangectasia: a human mutation giving high frequency misrepair of DNA double-stranded scissions. *Mol. Biol.. Med.*, **3**, 229–244.

DEBENHAM, P.G., WEBB, M.B.T., STRETCH, A. & THACKER, J. (1988*a*). Examination of vectors with two dominant selectable genes for DNA repair and mutation studies in mammalian cells. *Mutat. Res.*, **199**, 145–158.

DEBENHAM, P.G., JONES, N.J. & WEBB, M.B.T. (1988*b*). Vector-mediated DNA double-strand break repair analysisin normal, and radiation-sensitive, Chinese hamster V79 cells. *Mutat. Res.*, **199**, 1–9.

EVANS, H.L., RICANATI, M. & HORNG, M.-F. (1987). Deficiency of DNA repair in mouse lymphoma strain L5178Y-s. *Proc. Natl Acad. Sci. USA*, **84**, 7562–7566.

FOLGER, K.R., WONG, E.A., WAHL, G. & CAPECCHI, M.R. (1982). Patterns of integration of DNA microinjected into cultured mammalian cells: evidence for homologous recombination between injected plasmid DNA molecules. *Mol. Cell Biol.*, **2**, 1372–1387.

GIACCIA, A., WEINSTEIN, R. & STAMATO, T.D. (1985). Cell-cycle dependent repair of double-strand breaks in a gamma-ray sensitive Chinese hamster ovary cell. *Somat. Cell Genet.*, **11**, 485–491.

JEGGO, P.A. (1990). Studies on mammalian mutants defective in rejoining double-strand breaks in DNA. *Mutat. Res.*, **239**, 1–16.

JEGGO, P.A. & KEMP, L.M. (1983). X-ray-sensitive mutants of Chinese hamster ovary cell line: isolation and cross sensitivity to other DNA damaging agents. *Mutat. Res.*, **112**, 313–327.

JONES, N.J., COX, R. & THACKER, J. (1988). Six complementation groups for ionizing radiation sensitivity in Chinese hamster cells. *Mutat. Res.*, **193**, 139–144.

JONES, N.J., STEWART, S.A. & THOMPSON, L.H. (1990). Biochemical and genetic analysis of the Chinese hamster mutants *irs1* and *irs2* and their comparison to cultured ataxia telangiectasia cells. *Mutagenesis*, **5**, 15-23.

KEMP, L.H., SEDGWICK, S.G. & JEGGO, P.A. (1984). X-ray sensitive mutants of Chinese Hamster ovary cells defective in double strand break rejoining. *Mutat. Res.*, **132**, 189-196.

LEHMANN, A.R. (1982). The cellular and molecular responses of ataxia-telangiectasia cells to DNA damage. In *Ataxia-telangiectasia*, Bridges, B.A. & Harnden, D.G. (eds). pp. 379-401. John Wiley: New York.

MCMILLAN, T.J., CASSONI, A.M., EDWARDS, S., HOLMES, A. & PEACOCK, J.H. (1990). The relationship of DNA double strand break induction to radiosensitivity in human tumour cell lines. *Int. J. Radiat. Biol.*, **58**, 427-438.

MCMILLAN, T.J. & HOLMES, A. (1991). The isolation and partial characterization of a radiation sensitive clone of a human bladder carcinoma cell line. *Radiat. Res.*, **128**, 301-305.

MULLIGAN, R.C. & BERG, P. (1981). Selection for animal cells that express the Escherichia coli gene coding for xanthine-guanine phosphoribosyltransferase. *Proc. Natl Acad. Sci. USA*, **78**, 2072-2076.

OBE, G., VON DER HUDE, W., SCHEUTWINKEL-REICH, M. & BASLER, A. (1986). The restriction endonuclease AluI induces mutations in the HPRT locus but not in the Na+/K+ ATPase locus in V79 hamster cells. *Mutat. Res.*, **174**, 71-74.

PAINTER, R.B. (1981). Radioresistant DNA synthesis: an intrinsic feature of ataxia-telangiectasia. *Mutat. Res.*, **84**, 183-190.

POWELL, S.N. & MCMILLAN, T.J. (1991). Clonal variation of DNA repair for a human glioma cell line. *Radiother. Oncol.*, **21**, 225-232.

SATO, K. & HIEDA, N. (1979). Isolation and characterization of a mutant mouse lymphoma cell sensitive to methyl methanesulfonate and X-rays. *Radiat. Res.*, **78**, 167-171.

SCHWARTZ, J.L., ROTMENSCH, J., GIOVANAZZI, S., COHEN, M.B. & WEICHSELBAUM, R.R. (1988). Faster repair of DNA double-strand breaks in radioresistant human tumor cells. *Int. J. Radiat. Oncol. Biol. Phys.*, **15**, 907-912.

STAMATO, T.D. & DENKO, N. (1990). Assymetric field inversion gel electrophoresis: a new method for detecting DNA double strand breaks in mammalian cells. *Radiat. Res.*, **121**, 196-205.

WLODEK, D. & HITTELMAN, W.N. (1987). The repair of double-strand DNA breaks correlates with radiosensitivity of L5178Y-S and L5178Y-R cells. *Radiat. Res.*, **112**, 146-155.

WHITAKER, S.J. & MCMILLAN, T.J. (1992). Oxygen effect for DNA double-strand break induction determined by pulsed-field gel electrophoresis. *Int. J. Radiat. Biol.*, **61**, 29-41.


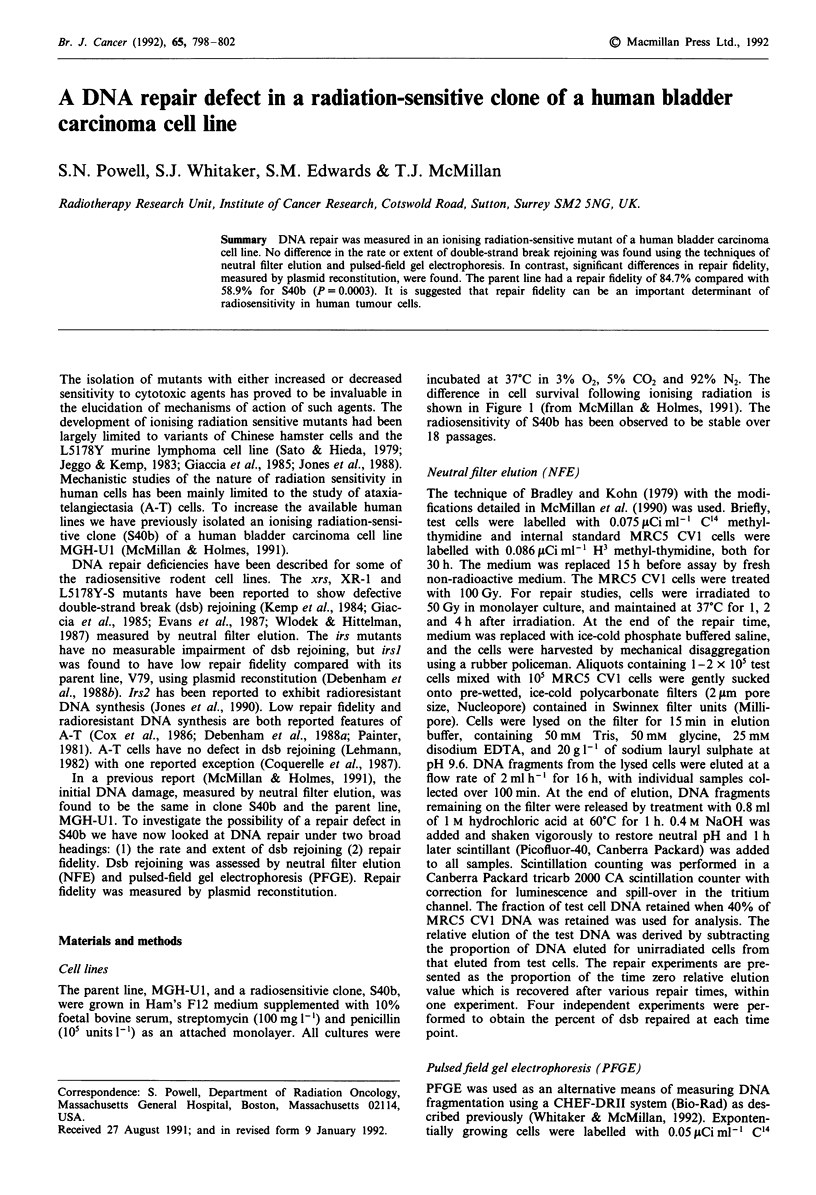

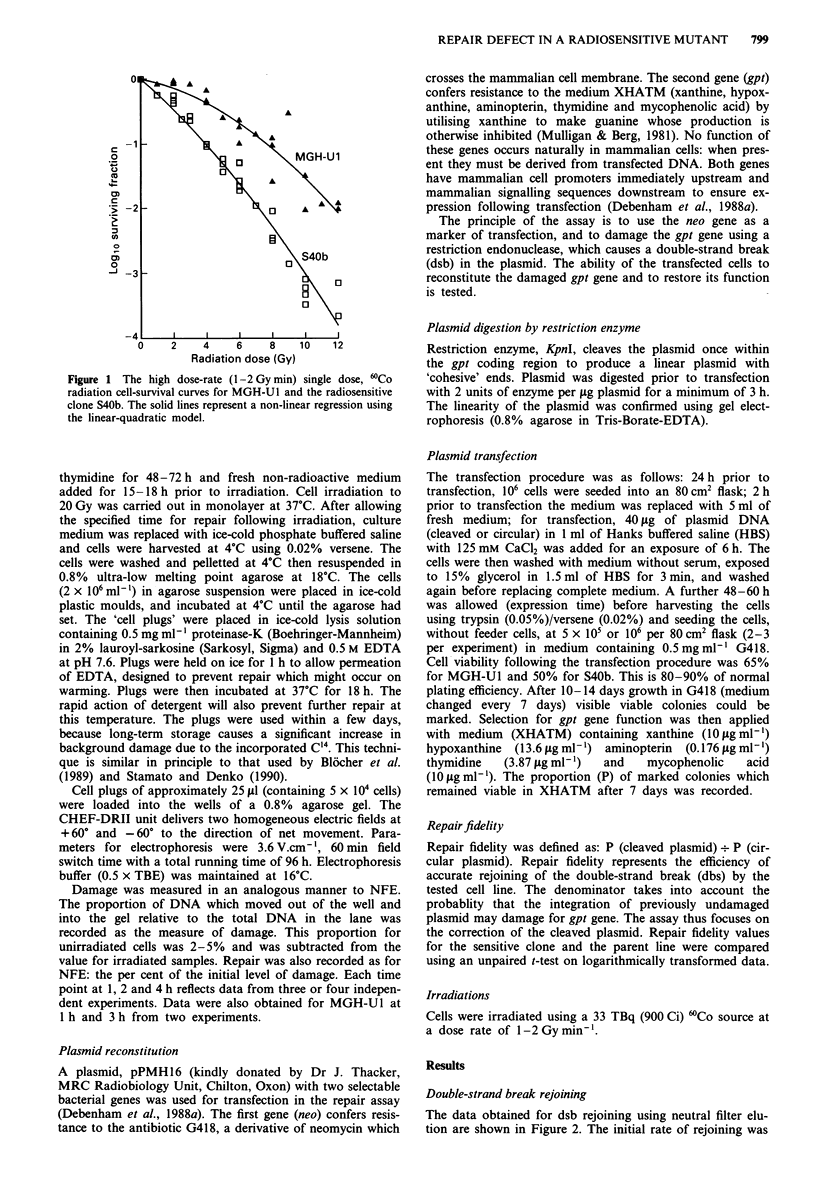

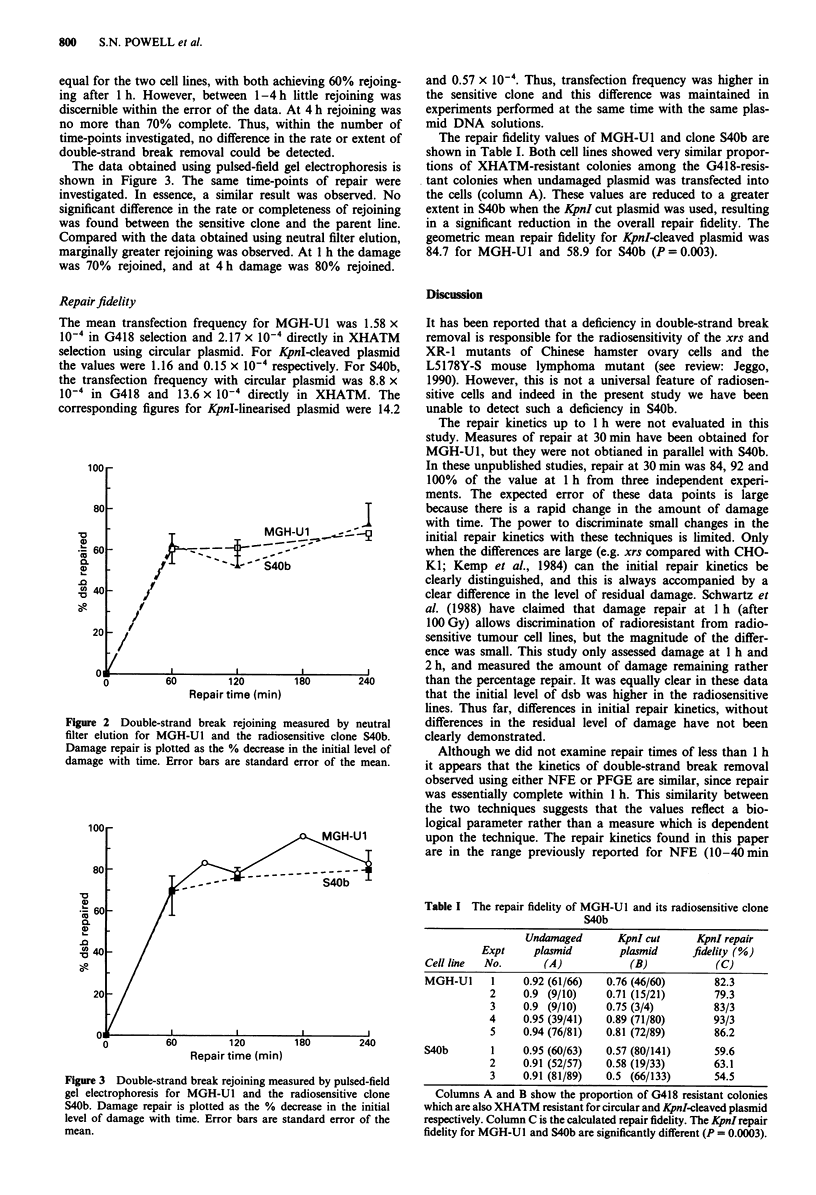

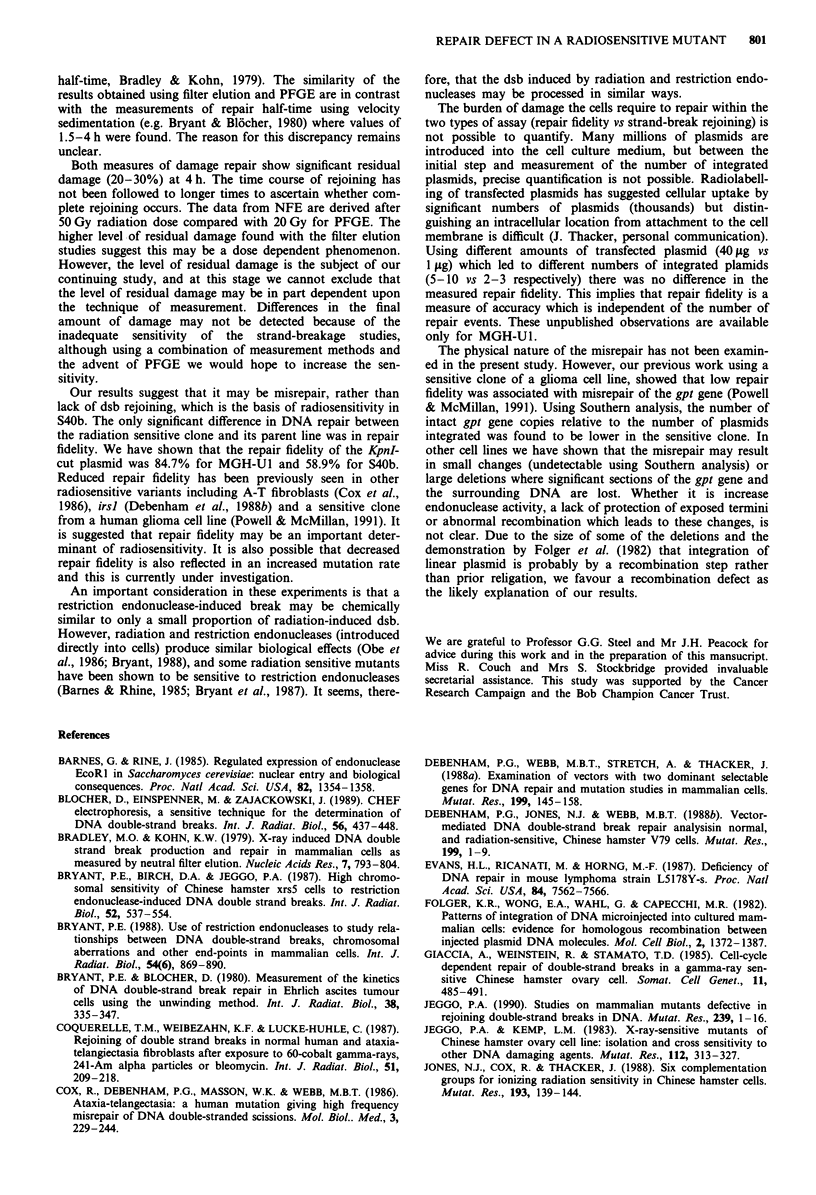

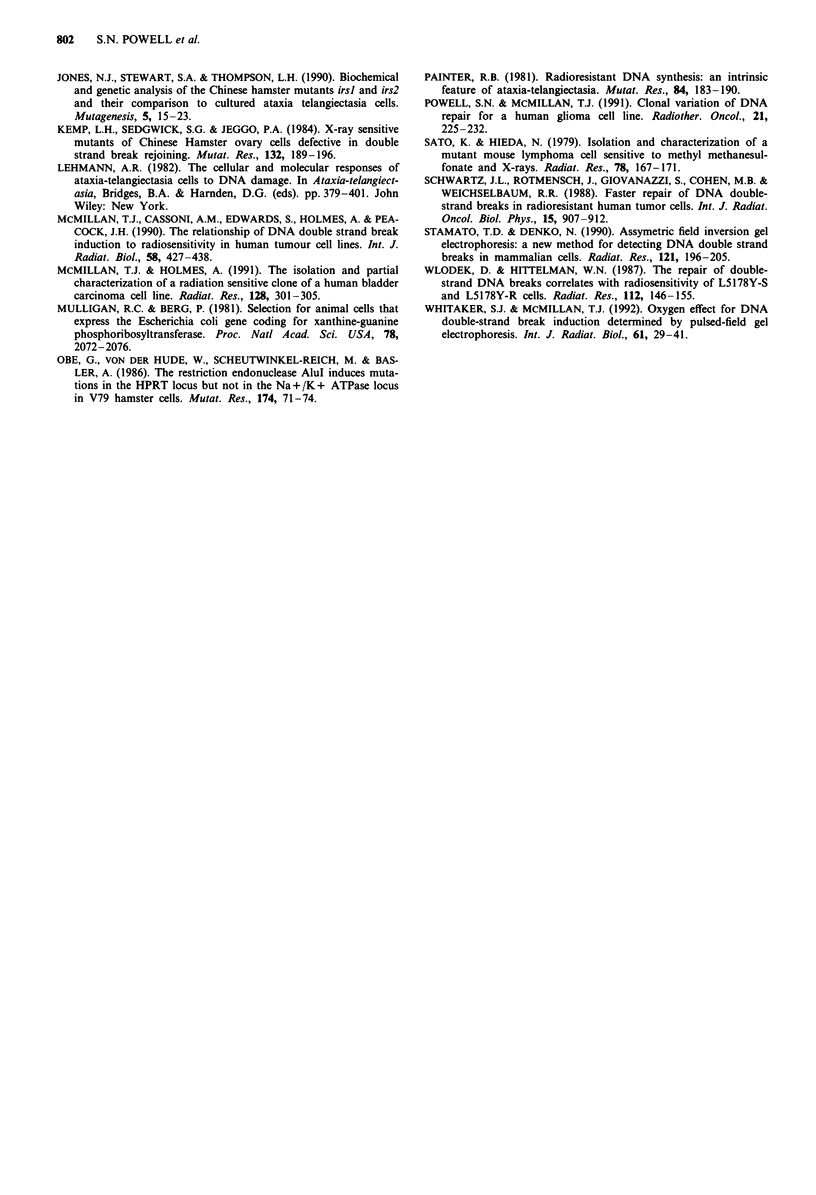


## References

[OCR_00573] Barnes G., Rine J. (1985). Regulated expression of endonuclease EcoRI in Saccharomyces cerevisiae: nuclear entry and biological consequences.. Proc Natl Acad Sci U S A.

[OCR_00578] Blöcher D., Einspenner M., Zajackowski J. (1989). CHEF electrophoresis, a sensitive technique for the determination of DNA double-strand breaks.. Int J Radiat Biol.

[OCR_00582] Bradley M. O., Kohn K. W. (1979). X-ray induced DNA double strand break production and repair in mammalian cells as measured by neutral filter elution.. Nucleic Acids Res.

[OCR_00586] Bryant P. E., Birch D. A., Jeggo P. A. (1987). High chromosomal sensitivity of Chinese hamster xrs 5 cells to restriction endonuclease induced DNA double-strand breaks.. Int J Radiat Biol Relat Stud Phys Chem Med.

[OCR_00598] Bryant P. E., Blöcher D. (1980). Measurement of the kinetics of DNA double strand break repair in Ehrlich ascites tumour cells using the unwinding method.. Int J Radiat Biol Relat Stud Phys Chem Med.

[OCR_00592] Bryant P. E. (1988). Use of restriction endonucleases to study relationships between DNA double-strand breaks, chromosomal aberrations and other end-points in mammalian cells.. Int J Radiat Biol.

[OCR_00604] Coquerelle T. M., Weibezahn K. F., Lücke-Huhle C. (1987). Rejoining of double strand breaks in normal human and ataxia-telangiectasia fibroblasts after exposure to 60Co gamma-rays, 241Am alpha-particles or bleomycin.. Int J Radiat Biol Relat Stud Phys Chem Med.

[OCR_00611] Cox R., Debenham P. G., Masson W. K., Webb M. B. (1986). Ataxia-telangiectasia: a human mutation giving high-frequency misrepair of DNA double-stranded scissions.. Mol Biol Med.

[OCR_00623] Debenham P. G., Jones N. J., Webb M. B. (1988). Vector-mediated DNA double-strand break repair analysis in normal, and radiation-sensitive, Chinese hamster V79 cells.. Mutat Res.

[OCR_00617] Debenham P. G., Webb M. B., Stretch A., Thacker J. (1988). Examination of vectors with two dominant, selectable genes for DNA repair and mutation studies in mammalian cells.. Mutat Res.

[OCR_00629] Evans H. H., Ricanati M., Horng M. F. (1987). Deficiency in DNA repair in mouse lymphoma strain L5178Y-S.. Proc Natl Acad Sci U S A.

[OCR_00634] Folger K. R., Wong E. A., Wahl G., Capecchi M. R. (1982). Patterns of integration of DNA microinjected into cultured mammalian cells: evidence for homologous recombination between injected plasmid DNA molecules.. Mol Cell Biol.

[OCR_00639] Giaccia A., Weinstein R., Hu J., Stamato T. D. (1985). Cell cycle-dependent repair of double-strand DNA breaks in a gamma-ray-sensitive Chinese hamster cell.. Somat Cell Mol Genet.

[OCR_00648] Jeggo P. A., Kemp L. M. (1983). X-ray-sensitive mutants of Chinese hamster ovary cell line. Isolation and cross-sensitivity to other DNA-damaging agents.. Mutat Res.

[OCR_00645] Jeggo P. A. (1990). Studies on mammalian mutants defective in rejoining double-strand breaks in DNA.. Mutat Res.

[OCR_00653] Jones N. J., Cox R., Thacker J. (1988). Six complementation groups for ionising-radiation sensitivity in Chinese hamster cells.. Mutat Res.

[OCR_00660] Jones N. J., Stewart S. A., Thompson L. H. (1990). Biochemical and genetic analysis of the Chinese hamster mutants irs1 and irs2 and their comparison to cultured ataxia telangiectasia cells.. Mutagenesis.

[OCR_00666] Kemp L. M., Sedgwick S. G., Jeggo P. A. (1984). X-ray sensitive mutants of Chinese hamster ovary cells defective in double-strand break rejoining.. Mutat Res.

[OCR_00679] McMillan T. J., Cassoni A. M., Edwards S., Holmes A., Peacock J. H. (1990). The relationship of DNA double-strand break induction to radiosensitivity in human tumour cell lines.. Int J Radiat Biol.

[OCR_00683] McMillan T. J., Holmes A. (1991). The isolation and partial characterization of a radiation-sensitive clone of a human bladder carcinoma cell line.. Radiat Res.

[OCR_00688] Mulligan R. C., Berg P. (1981). Selection for animal cells that express the Escherichia coli gene coding for xanthine-guanine phosphoribosyltransferase.. Proc Natl Acad Sci U S A.

[OCR_00696] Obe G., Von der Hude W., Scheutwinkel-Reich M., Basler A. (1986). The restriction endonuclease Alu I induces chromosomal aberrations and mutations in the hypoxanthine phosphoribosyltransferase locus, but not in the Na+/K+ ATPase locus in V79 hamster cells.. Mutat Res.

[OCR_00700] Painter R. B. (1981). Radioresistant DNA synthesis: an intrinsic feature of ataxia telangiectasia.. Mutat Res.

[OCR_00704] Powell S., McMillan T. J. (1991). Clonal variation of DNA repair in a human glioma cell line.. Radiother Oncol.

[OCR_00709] Sato K., Hieda N. (1979). Isolation and characterization of a mutant mouse lymphoma cell sensitive to methyl methanesulfonate and X rays.. Radiat Res.

[OCR_00714] Schwartz J. L., Rotmensch J., Giovanazzi S., Cohen M. B., Weichselbaum R. R. (1988). Faster repair of DNA double-strand breaks in radioresistant human tumor cells.. Int J Radiat Oncol Biol Phys.

[OCR_00720] Stamato T. D., Denko N. (1990). Asymmetric field inversion gel electrophoresis: a new method for detecting DNA double-strand breaks in mammalian cells.. Radiat Res.

[OCR_00730] Whitaker S. J., McMillan T. J. (1992). Oxygen effect for DNA double-strand break induction determined by pulsed-field gel electrophoresis.. Int J Radiat Biol.

[OCR_00725] Wlodek D., Hittelman W. N. (1987). The repair of double-strand DNA breaks correlates with radiosensitivity of L5178Y-S and L5178Y-R cells.. Radiat Res.

